# Aurora-A contributes to cisplatin resistance and lymphatic metastasis in non-small cell lung cancer and predicts poor prognosis

**DOI:** 10.1186/1479-5876-12-200

**Published:** 2014-07-31

**Authors:** Jie Xu, Cai-feng Yue, Wei-hua Zhou, Yuan-min Qian, Yan Zhang, Shao-wu Wang, An-wen Liu, Quentin Liu

**Affiliations:** 1 State Key Laboratory of Oncology in South China, Cancer Center, Sun Yat-sen University, Guangzhou 510060, China; 2 Institute of Cancer Stem Cell, Dalian Medical University, Dalian 116044, China; 3 Department of Oncology, the Second Affiliated Hospital, Nanchang University, 1 Minde Road, Nanchang 330006, China; 4 Department of Radiology, The First Affiliated Hospital, Dalian Medical University, Dalian 116044, China

**Keywords:** Non-small cell lung cancer, Aurora-A, Cisplatin resistance, Prognosis, Metastasis

## Abstract

**Background:**

Platinum-based chemotherapy improves survival among patients with non-small cell lung cancer (NSCLC), but the efficiency is limited due to resistance. In this study, we aimed to identify the expression of Aurora-A and its correlation with cisplatin resistance and prognosis in NSCLC.

**Methods:**

We used immunohistochemical analysis to determine the expression of Aurora-A protein in 102 NSCLC patients treated by surgery and adjuvant cisplatin-based chemotherapy. The prognostic significances were assessed by Kaplan-Meier survival estimates and Cox models. The potential role of Aurora-A in the regulation of cisplatin resistance in NSCLC cells was examined by transfections using expression vector and small interfering RNA or using small-molecule inhibitors.

**Results:**

Aurora-A expression was significantly associated with clinical stage (*p* = 0.018), lymph node metastasis (*p* = 0.038) and recurrence (*p* = 0.005), and was an independent prognostic parameter in multivariate analysis. High level of Aurora-A expression predicted poorer overall survival (OS) and progression-free survival (PFS). *In vitro* data showed that Aurora-A expression was elevated in cisplatin-resistant lung cancer cells, and overexpression or knockdown of Aurora-A resulted in increased or decreased cellular resistance to cisplatin. Furthermore, inhibition of Aurora-A reversed the migration ability of cisplatin-resistant cells.

**Conclusions:**

The current findings suggest that high Aurora-A expression is correlated with cisplatin-based chemotherapeutic resistance and predicts poor patient survival in NSCLC. Aurora-A might serve as a predictive biomarker of drug response and therapeutic target to reverse chemotherapy resistance.

## Background

Lung cancer is the leading cause of cancer mortality worldwide [1]. Non-small cell lung cancer (NSCLC) accounts for approximately 85% of lung cancers, including squamous cell carcinoma, adenocarcinoma and large cell carcinoma [2]. The overall survival for NSCLC remains poor, with 5-year survival rates only 10–20% [3]. Currently, surgery represents the main curative treatment modality for cure of NSCLC. Additional therapy is necessary because of high rates of distant and local disease recurrence after surgical resection. Platinum-based chemotherapy (cisplatin or carboplatin in combination with navelbine, gemcitabine, or paclitaxel) has been widely used as first-line regimen for advanced NSCLC. However, NSCLC patients frequently exhibit resistance to chemotherapy [4,5]. Previous studies have revealed that the major resistance mechanisms include reduced uptake and/or increased efflux, enhanced DNA repair, and failure of cell-death pathways, metastatic tumor [6]. In addition, several studies have reported multiple genetic aberrations that might predict cisplatin sensitivity in NSCLC patients, such as ERCC1, BRCA1, XRCC1 and CFL1 [7-9]. However, much work remains to be done to validate the clinical relevance of resistance genes to benefit from adjuvant platinum-based chemotherapy.

Aurora-A (also called STK15/BTAK) is a member of serine/threonine kinase family. It is involved in various mitotic events, such as centrosome maturation and separation, mitotic entry, bipolar-spindle assembly, chromosome alignment on the metaphase plate and cytokinesis. In human cells, Aurora-A expression and kinase activity are increased during late G2 to M phase, and its subcellular localization dynamically changes during the cell cycle [10]. The levels of Aurora-A mRNA and protein are increased in various malignant tumors including colon, breast, bladder, ovarian and pancreatic cancers, indicating that it is important for tumor formation or progression [11-14]. Furthermore, we and others have previously showed that overexpression of Aurora-A increases migration and leads to resistance to chemotherapy drugs [15-18]. Accordingly, Aurora-A serves as a promising target in cancer therapy, and several small-molecule inhibitors for Aurora-A kinase are currently being investigated within clinical trials [19]. The novel Aurora-A kinase inhibitor alisertib (MLN8237) is currently undergoing evaluation in a phase I/II trial with paclitaxel in recurrent ovarian cancer [20]. The inhibitor alisertib seems clinically active in both B- and T-cell aggressive lymphomas, and confirmatory single-agent and combination studies have been initiated [21].

In the present study, we detected the expression of Aurora-A in NSCLC patients treated with adjuvant cisplatin-based therapy and determined its correlation with clinical characteristics. We also validated an association between elevated Aurora-A expression and cisplatin resistance in vitro. In addition, we investigated the effect of Aurora-A on the sensitivity of cisplatin in A549 and H460 human lung cancer cells.

## Methods

### Patients and clinical specimens

The present study included 102 patients with NSCLC, diagnosed at the Cancer Center, Sun Yat-sen University between 2002 and 2003. The patients, 70 males (69%) and 32 females (31%), ranged in age from 38 to 75 years (mean 60 years). Histological examination was performed on formalin-fixed tissues in all cases and tumors were diagnosed and classified according to the AJCC (American Joint Committee on Cancer guidelines) classification [22].

All the patients (from stage I to stage III) underwent radical surgery of primary tumor and lymph nodes, and received adjuvant cisplatin-based chemotherapy after surgery. No chemotherapy or radiotherapy was given to patients before surgery. Written informed consent for the use of the tissues was obtained from all patients before surgery, and the study was approved by the Institute Research Ethics Committee of Sun Yat-sen University.

### Cell lines and cell culture

The human lung adenocarcinoma cell lines A549 and A549/DDP (cisplatin-resistant variant) were graciously provided by Dr. Xiaofeng Zhu, Sun Yat-sen University [23]. The lung cancer cell lines NCI-H460 (H460) and H460/DDP (cisplatin-resistant variant) were graciously provided by Dr. Liwu Fu, Sun Yat-sen University [24]. Both cell lines were cultured in RPMI-1640 supplemented with 10% fetal bovine serum at 37°C in humidified 5% CO_2_ incubator. The A549/DDP and H460/DDP cells were cultured with 6 μM cisplatin (Sigma–Aldrich, St.Louis, MO) to maintain drug resistance.

### Transient transfection

Cells were transfected with an empty vector pCS2+ or pCS2 + -Aurora-A (a gift from Joan Ruderman, Harvard Medical School, Boston, MA), or interfering RNA (siRNA) and negative control using Lipofectamine 2000 as described earlier [17]. The negative control (NC) siRNA and siRNA against Aurora-A (5′-AUGCCCUGUCUUACUGUCA-3′) were synthesized by GenePharma Company (Shanghai).

### Lentiviral transfection for stable expression cells

Plasmid pLVX-DsRed-N1-Monomer (Clontech) expressing Aurora-A was from Dr. Feimeng Zheng, Sun Yat-sen University. The shRNA sequences against GFP (Sence-5′-GCAAGCTGACCCTGAAGTTCAT, Antisense-5′-ATGAACTTCAGGGTCAGCTTGC) and Aurora-A (Sence-5′-CACATACCAAGAGACCTACAA, Antisense-5′-TTGTAGGTCTCTTGGTATGTG) were cloned into pLKO-Tet-On (Addgene). Lentiviruse was produced in 293T cells as described earlier [25].

### Cell viability assay

The MTT assay was used to assess cell viability. 5,000 cells were plated in 96-well plates and then were exposed to various concentrations of cisplatin, VX-680 (Kava Technology), MLN8237 (Selleck Chemicals) or combinations. Following treatment, 0.5 mg/ml MTT (Sigma–Aldrich) solution was added to each well, and incubated for 4 hours (h). After the incubation, culture media was discarded followed by addition of 0.15 ml DMSO and vibration for 10 minutes (min). The absorbance at 570 nm was determined. The cell inhibition ratio was calculated as a fraction of the untreated controls.

### Colony formation assay

5,000 cells were seeded into six-well plates in triplicate and incubated for 8–10 days. Colonies were stained with crystal violet and counted.

### Western blot analysis

Cells were lysed with the RIPA buffer on ice before being subjected to western blot analysis. The protein concentration was detected by the Bradford method. Total proteins were fractionated using SDS-PAGE and transferred onto nitrocellulose membrane. The membrane was blocked and incubated with mouse anti-GAPDH antibody (Ambion Biotechnology), rabbit anti-Aurora-A antibody (Upstate), rabbit anti-p-Aurora-A antibody (Thr288, Cell Signaling), rabbit anti-histone H3 antibody (Epitomics), and rabbit anti-p-histone H3 antibody (Ser10, SAB).

### Immunofluorescence staining

Cells were fixed with 4% paraformaldehyde at room temperature for 15 min and permeabilized in 0.25% Triton X-100 in PBS for 10 min, and incubated in 3% BSA blocking solution for 30 min. The cells were incubated with anti-Aurora-A (Upstate) and α-tubulin (Sigma) antibodies for 1 h. The immune complexes were detected with goat anti-mouse conjugated to Alexa-488 and goat anti-rabbit conjugated to Alexa-546 secondary antibody (Molecular Probes). Nucleus was stained with DAPI.

### Flow cytometry

Apoptosis analysis was conducted with an Annexin V-FITC Apoptosis Detection Kit (KeyGen Biotech) according to the manufacturer’s protocol. The percentage of apoptotic cells was determined using FACS flow cytometer equipped software (BECKMAN).

### Wound healing assay

Cells transfected with RNAi or not were seeded in 24-well plates and grown until 70-80% confluence. The cells were then serum starved for 24 h. A linear wound was created using a pipette tip and observed and photographed at various times as indicated in the figure legends.

### Immunohistochemistry analysis and evaluation

Formalin-fixed, paraffin-embedded samples were cut in 4 μm sections and mounted on slides. Slides were deparaffinized, rehydrated, and treated with 3% H_2_O_2_ in methanol for 15 min to inhibit endogenous peroxidase. Pretreatment was done in microwave with Tris/EDTA buffer solution (pH 8.0). After transfer to a humidified chamber, the slides were blocked with 10% normal goat serum at room temperature for 30 min and incubated with mouse anti-Aurora-A monoclonal antibody (Sigma, A1231, 1:200 dilution). Detection of the primary antibody was done using the Envision DAKO EnVision Detection kit, (3, 3-diaminobenzidine; code K 5007, DAKO Cytomation, Glostrup, Denmark) according to the protocol of the manufacturer. Finally, samples were counterstained with hematoxylin, dehydrated, and mounted. The positive control sample was a normal colonic mucosa section known to express Aurora-A. The negative control was obtained by replacing the primary antibody with a normal murine IgG.

Each case was rated according to a score that added a scale of intensity of staining to the area of staining. At least 10 high-power fields were chosen randomly, and >1,000 cells were counted for each section. The intensity of staining was graded on the following scale: 0, no staining; 1, weak; 2, moderate; 3, strong. The area of staining was evaluated as follows: 0, no staining of cells in any microscopic fields; 1+, <30% of tissue stained positive; 2+, between 30% and 60% stained positive; 3+, >60% stained positive [26]. The minimum score when summed (extension + intensity) was therefore, 0, and the maximum. Two independent pathologists (W.H.Z. and J.X.), blind to follow-up data, were responsible for IHC staining evaluation. A third pathologist arbitrated when any discrepancy arose between these two pathologists.

### Selection of cutoff score for Aurora-A expression

Receiver operating characteristic (ROC) curve analysis was carried out to select Aurora-A cutoff score [27,28]. In brief, at each immunohistochemical score, the sensitivity and specificity for the outcome under study was plotted, generating an ROC curve. The score localized closest to the point at both maximizing sensitivity and specificity, the point (0.0, 1.0) on the curve, was selected as the cutoff score above which Aurora-A expression was considered high. To facilitate ROC curve analysis, the survival features were dichotomized: survival (death VS. others (censored, alive or death from other causes)).

### Follow up

All patients had follow-up records for over 5 years. After the completion of therapy, patients were observed at 3 month intervals during the first 3 years and at 6 month intervals thereafter. OS was defined as the time from diagnosis to the date of death or when censored at the latest date if patients were still alive. PFS was assessed from the first day of treatment to the earliest signs of disease progression as determined by CT or MRI imaging using RECIST (Response Evaluation Criteria In Solid Tumors) criteria, or death from any cause.

### Statistical analysis

Optimal cutpoint for Aurora-A expression was obtained by ROC analysis. The chi-square test or Fisher’s exact test was used to estimate the correlation between Aurora-A expression and clinic pathological variables. Univariate survival analysis for Aurora-A was carried out using the Kaplan-Meier method, and the differences in survival probabilities were evaluated by the log-rant test. The hazard ratios and 95% confidence intervals for patient outcome were determined using the multivariate Cox proportional hazards model. Statistical analysis was performed using SPSS v. 17.0 (SPSS, Inc, Chicago, IL). Two-sided P values < 0.05 were considered statistically significant. Experiments were repeated at least three times. Data were mean ± SD. Differences among variables were evaluated by ANOVA, two-tailed Student’s *t*-tests. P values < 0.05 were considered to be significant.

## Results

### Aurora-A expression in NSCLC tissues

**Figure 1:**
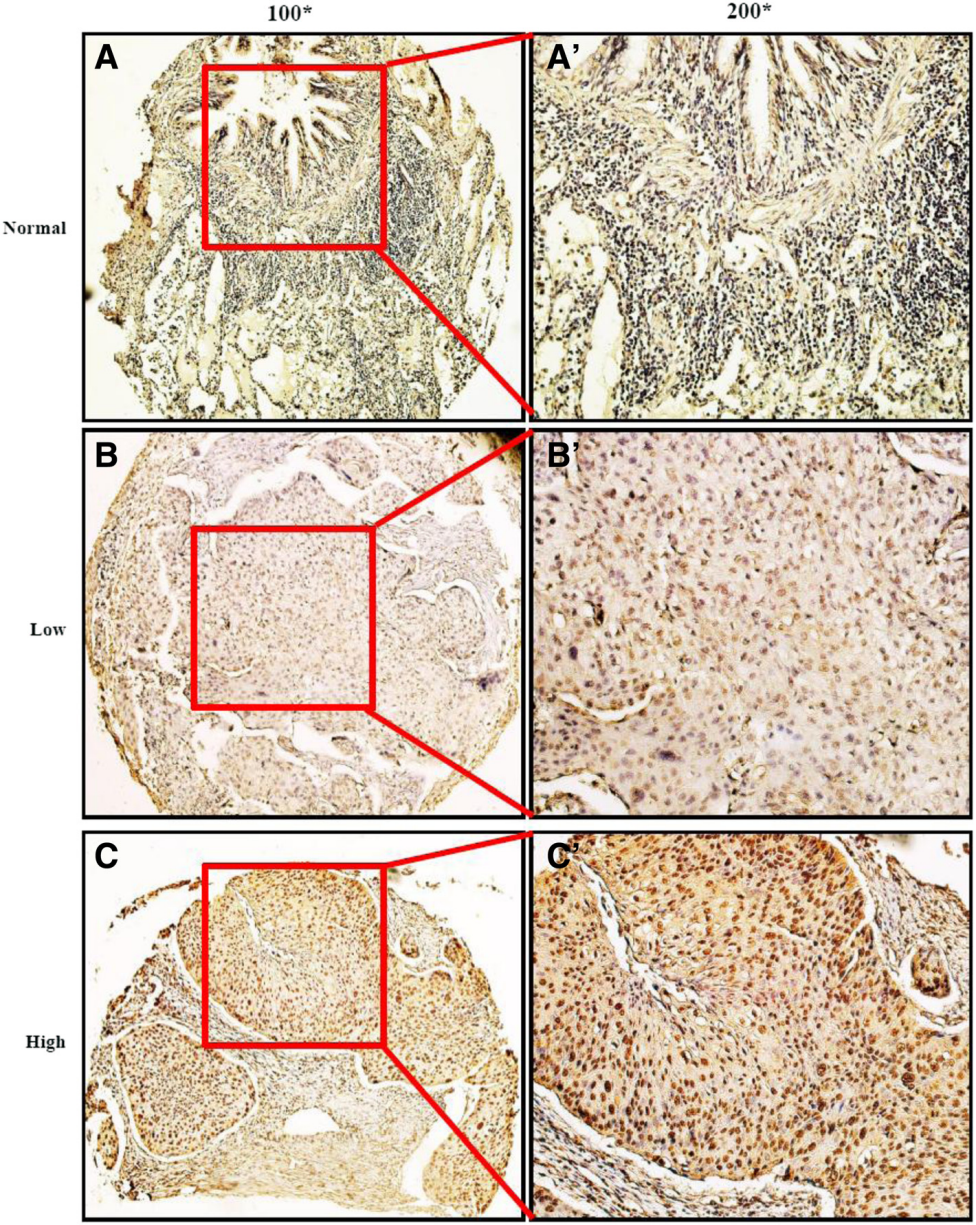
**Representative specimens of Aurora-A expression are shown in tissue microarrays by immunohistochemistry analysis. (A)** Non-neoplastic lung tissues showed nearly negative expression of Aurora-A (100×). **(B)** Low-intensity Aurora-A expression was shown in a NSCLC patient sample (100×). **(C)** High-intensity Aurora-A expression was shown in another NSCLC case (100×). **(A’)**, **(B’)**, **(C’)** demonstrated the higher magnification (200×) from the area of the box in **(A)**, **(B)**, **(C)** respectively.

The specificity for antibody targeting Aurora-A was showed in Additional file 1: Figure S1 by western blot analysis. The staining of Aurora-A was nearly negative (Figure 1A and A’) in adjacent non-neoplastic lung tissues, whereas NSCLC tissues showed elevated expression of Aurora-A (Figure 1B and C). Aurora-A staining located in both nucleus and cytoplasm, predominantly in nucleus (Figure 1B’ and C’). To select a relevant immunohistochemical cutoff score to describe Aurora-A overexpression in NSCLC, receiver operating characteristic (ROC) curve analysis was carried out. As shown in Additional file 1: Figure S2, the Aurora-A cutoff scores for OS and PFS were 3.4 (*p* = 0.004) and 3.2 (*p* = 0.014) respectively. We thus divided the cohort into high (score ≥ 4) and low (score < 4) populations based on the cutoff points.

### Aurora-A expression and clinicopathological characteristics

Clinicopathological features were listed in Table 1 in relation to Aurora-A expression status. High expression of Aurora-A was detected in 57 out of 102 (55.9%) selected NSCLC tissues and low in other 45 (44.1%) cases. In high-Aurora-A expression group, the incidence of cases in stage III was statistically significantly higher than in the low-Aurora-A expression group (77.2% vs. 51.1%, respectively; *p* = 0.006). Further correlation analysis demonstrated that high Aurora-A expression was significantly associated with clinical stage (*p* = 0.018), lymph node metastasis (*p* = 0.038) and recurrence (*p* = 0.005). Regarding other clinicopathological variables, there was no statistically significant correlation observed in age, gender, smoking history, carcino-embryonic antigen (CEA), histology, differentiation and tumor stage.

**Table 1:** Association of Aurora-A expression with patient’s clinicopathologic characteristics in NSCLC

		**Aurora-A**		
**Variable**	**All cases**	**High**	**Low**	***P***
**Age (years)**				
≥ 60.0	36	20	16	
< 60.0	66	37	29	0.961
**Gender**				
Male	70	35	35	
Female	32	22	10	0.077
**Smoking history**				
Yes	57	30	27	
No	45	27	18	0.457
**CEA (ng/ml)**				
> 5.0	52	32	20	
≤ 5.0	50	25	25	0.241
**Initial clinical stage**				
I	11	5	6	
II	24	8	16	0.018
III	67	44	23	
**Histology**				
Squamous cell carcinoma	12	7	5	
Adenocarcinoma	88	48	40	0.433
Adenosquamous cell carcinomas	2	2	0	
**Differentiation**				
Highly	15	6	9	
Modrately	33	17	16	0.236
Poorly	54	34	20	
**Tumor stage**				
T_1_ + T_2_	63	34	29	
T_3_ + T_4_	39	23	16	0.621
**Lymph node metastasis**				
Negative	26	10	16	
Positive	76	47	29	0.038
**Recurrence**				
Local	35	15	20	
Distance	46	36	10	0.005
Negative	21	6	15	

### Aurora-A expression and survival

**Figure 2:**
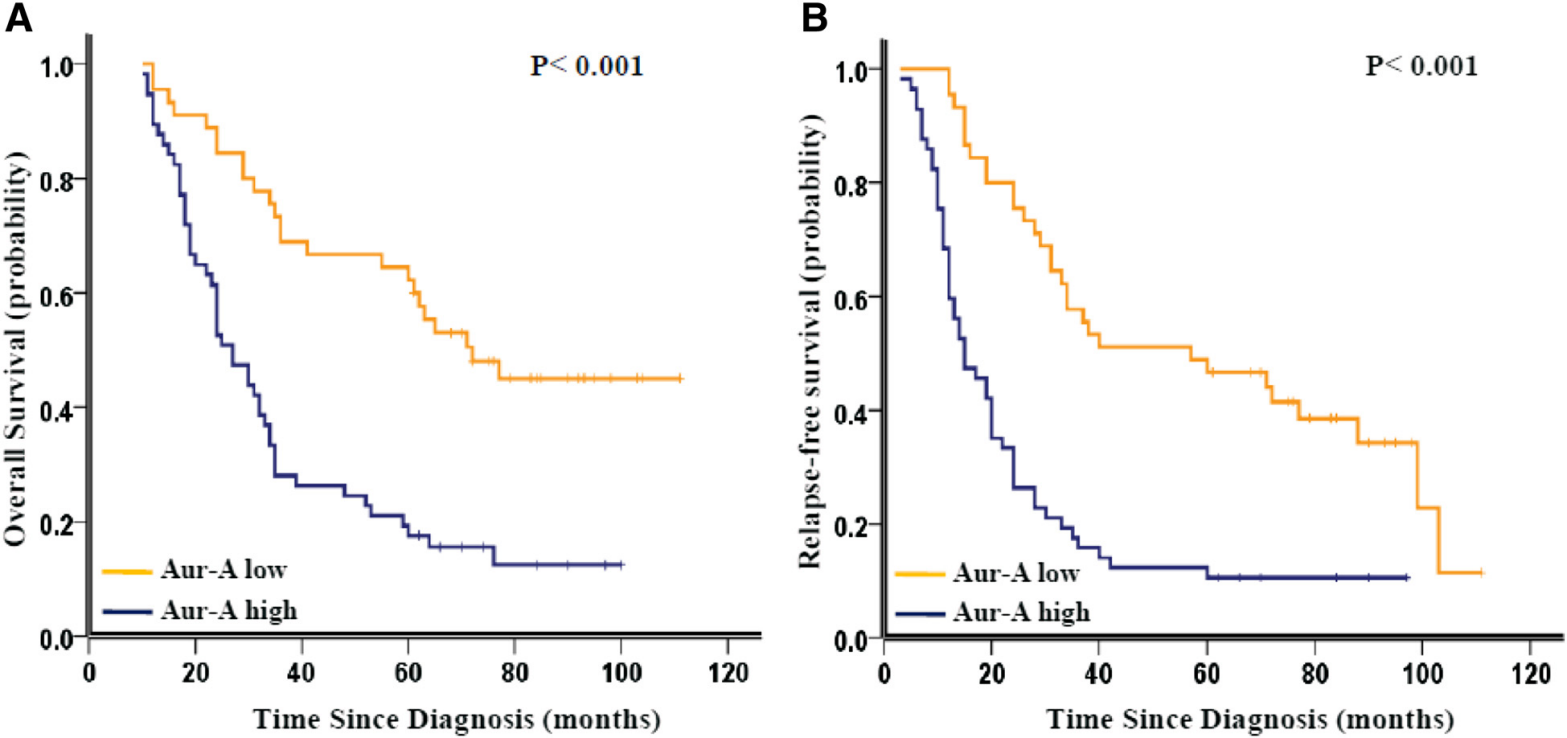
**Kaplan-Meier survival analysis of Aurora-A expression in NSCLC patients. (A)** High expression of Aurora-A (Aur-A) was closely correlated with poor overall survival, **(B)** and progression-free survival. The median duration of overall survival for patients with high and low expression of Aurora-A was 27.25 vs. 72.2 months (*p* < 0.001) and 15.5 vs. 57.5 months (*p* < 0.001), respectively.

The Kaplan–Meier analysis indicated that the overall survival (OS) of patients with high Aurora-A expression was significantly poorer than those with low Aurora-A expression (the median duration of OS 27.25 vs. 72.2 months, respectively, *p* < 0.001; Figure 2A). Also, elevated expression of Aurora-A predicted an inferior progression-free survival (PFS) with the median duration of PFS 15.5 vs. 57.5 months respectively (*p* < 0.001, Figure 2B).

**Figure 3:**
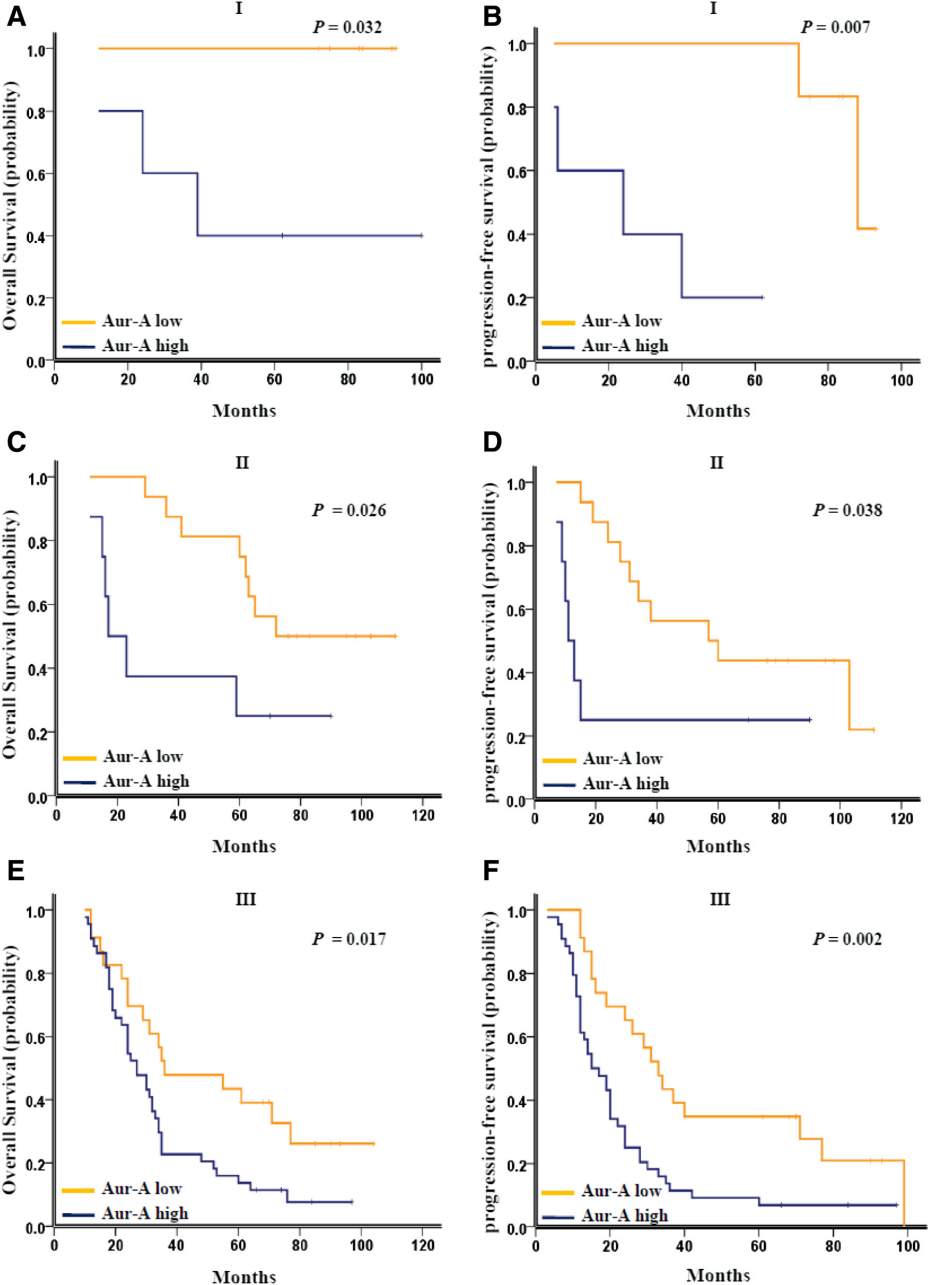
**Kaplan-Meier survival analysis of Aurora-A expression in subsets of NSCLC patients with different stage. (A)** Probability of overall survival and **(B)** progression-free survival of NSCLC patients with stage I in the testing set: low expression, n = 6; high expression, n = 5. **(C)** Probability of overall survival and **(D)** progression-free survival of NSCLC patients with stage II in the testing set: low expression, n = 16; high expression, n = 8. **(E)** Probability of overall survival and **(F)** progression-free survival of NSCLC patients with stage III in the testing set: low expression, n = 23; high expression, n = 44.

Further analysis was performed between Aurora-A expression and subsets of NSCLC patients with each clinical stage. High expression of Aurora-A associated with a significant trend toward worse OS and PFS in patients of stage I (*p* = 0.032 for OS and *p* = 0.007 for PFS, Figure 3A and B), stage II (*p* = 0.026 for OS and *p* = 0.038 for PFS, Figure 3C and D), and stage III (*p* = 0.017 for OS and *p* = 0.002 for PFS, Figure 3E and F).

### Multivariate Cox regression analysis

Multivariate analysis using the Cox proportional hazards model revealed that high Aurora-A expression was an independent and significant prognostic factor for OS (hazard ratio: 3.311; 95%CI: 1.899-5.775; *p* < 0.001; Table 2) and PFS (hazard ratio: 3.360; 95%CI: 2.066-5.466; *p* = 0.003; Additional file 2: Table S1). Of other parameters, CEA level was evaluated as a positive independent prognostic factor for OS and PFS, tumor size for PFS.

**Table 2:** Results of univariate and multivariate Cox proportional-hazards analysis in the overall patient for death

	**For death**					
**Variable**	**Univariate analysis**			**Multivariate analysis**		
	**Hazard ratio**	**95% confidence interval**	***P***	**Hazard ratio**	**95% confidence interval**	***P***
**Age (years) ≥ 60.00 (VS. < 60.0)**	1.186	(0.734 to 1.916)	0.487	1.016	(0.603 to 1.712)	0.952
**Gender Male (VS. Female)**	1.249	(0.752 to 2.074)	0.391	1.057	(0.451 to 2.477)	0.899
**Smoking history Yes (VS. No)**	1.504	(0.941 to 2.406)	0.088	1.520	(0.930 to 2.458)	0.095
**CEA (ng/ml) > 5 (VS. ≤ 5)**	2.070	(1.288 to 3.326)	0.003	2.034	(1.223 to 3.385)	0.006
**Initial clinical stage**						
I	0.187	(0.058 to 0.601)	0.005	0.281	(0.086 to 0.917)	0.035
II	0.449	(0.248 to 0.812)	0.008	0.724	(0.384 to 1.364)	0.317
III	1	1		1	1	
**Histology**						
Squamous cell carcinoma	1.898	(0.263 to 13.700)	0.525	5.540	(0.749 to 40.949)	0.093
Adenocarcinoma	2.191	(0.277 to 17.325)	0.457	5.725	(0.688 to 47.634)	0.107
Adenosquamous cell carcinomas	1	1		1	1	
**Differentiatiation**						
Highly	0.581	(0.281 to 1.200)	0.142	1.138	(0.514 to 2.521)	0.750
Moderately	0.796	(0.479 to 1.322)	0.377	0.993	(0.562 to 1.757)	0.982
Poorly	1	1		1	1	
**Tumor stage T**_**4**_ **+ T**_**3 **_**(VS. T**_**2**_ **+ T**_**1**_**)**	1.534	(0.962 to 2.445)	0.072	1.295	(0.752 to 2.230)	0.352
**Lymph node metastasis Positive (VS. Negative)**	2.460	(1.319 to 4.588)	0.005	1.484	(0.519 to 4.247)	0.462
**Aurora-A High (VS. Low)**	2.971	(1.803 to 4.895)	0.000	3.311	(1.899 to 5.775)	0.000

### Overexpression of Aurora-A increases cellular resistance to cisplatin

**Figure 4:**
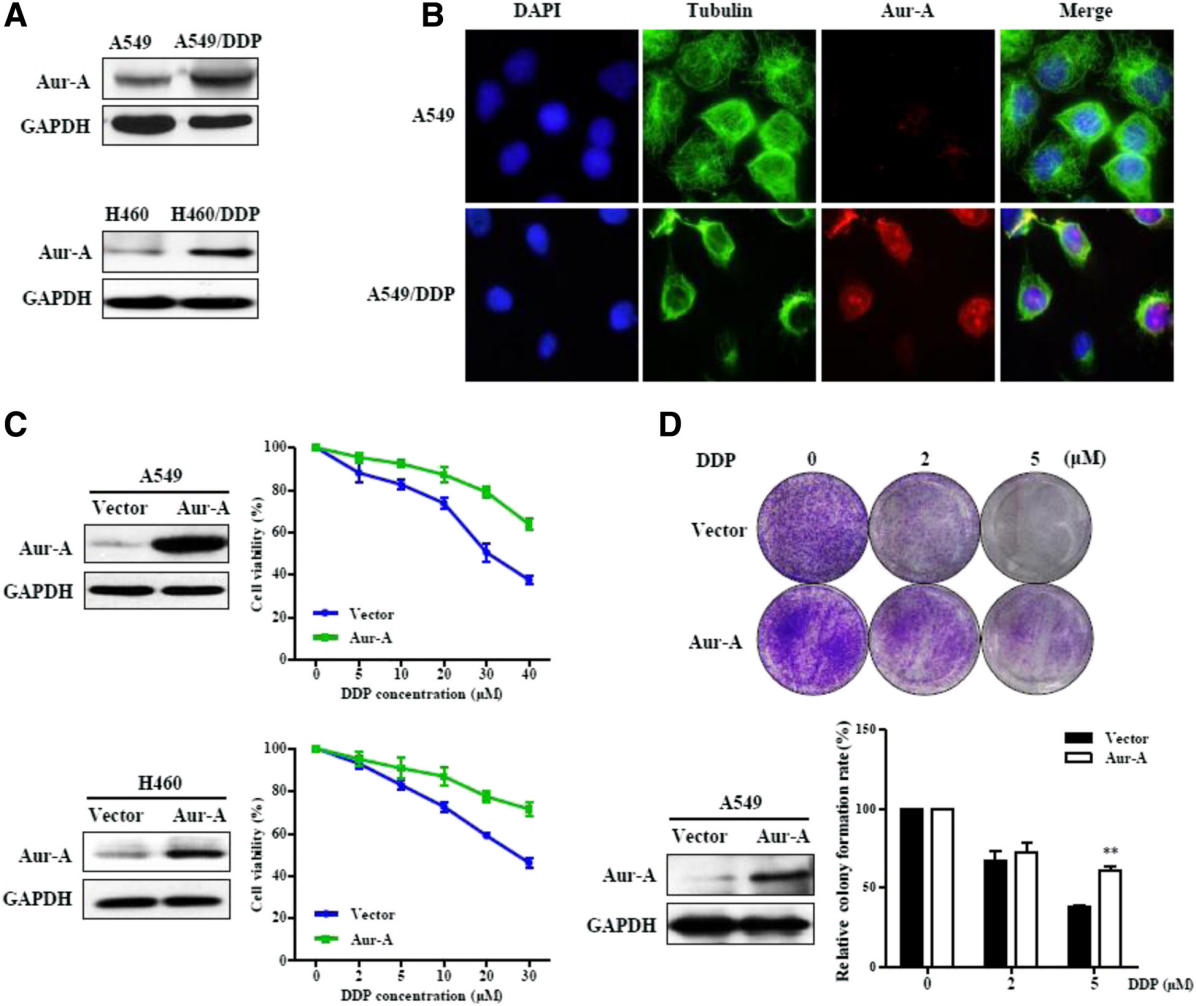
**Aurora-A overexpression correlates with cisplatin resistance in vitro. (A)** Protein level of Aurora-A was detected in A549 and A549/DDP cells or in H460 and H460/DDP cells by western blotting. Equal loading of protein was determined by GAPDH. **(B)** The staining of Aurora-A by Immunofluorescence in A549 and A549/DDP cells. Red: Aurora-A; Green: Tubulin; Blue: DAPI. **(C)** A549 and H460 cells were transfected with an expression vector for Aurora-A or control (vector) and detected by western blotting (left panel). Cells were treated with indicated concentrations of DDP for 24 h and subjected to MTT assay (right panel). **(D)** Aurora-A was overexpressed in A549 cells through lentivirus-mediated gene transfer. Two days after transfection, cells were selected by using 2 μg/ml puromycin. Cells were harvested for western blotting analysis and subjected to colony formation assay with incubation of DDP (2, 5 μM) or vehicle (0 μM) (n = 3), **p < 0.01, two-tailed Student’s t-tests.

Since high level of Aurora-A was correlated with poor prognosis in NSCLC patients that treated by platinum-based chemotherapy, we sought to explore the association between Aurora-A expression and cisplatin-resistance in vitro. We detected the protein expression level of Aurora-A in A549 and drug-resistant A549/DDP cells by Western blot and immunofluorescence analysis. As shown in Figure 4A (upper panel) and B, Aurora-A expression was elevated in cisplatin-resistant A549/DDP cells. A549/DDP cells with higher Aurora-A level also showed raised proliferation (Additional file 1: Figure S3). In another lung cancer drug-resistant cell line H460/DDP, Aurora-A expression was elevated relative to H460 cells (Figure 4A, lower panel). Then we performed forced expression of Aurora-A in A549 and H460 cells (Figure 4C), and treated the cells with increasing concentrations of cisplatin. Forced expression of Aurora-A increased the resistance of A549 and H460 cells to cisplatin in cell viability assay. Colony formation assay indicated that overexpression of Aurora-A enhanced cell growth and resistance to cisplatin in A549 cells (Figure 4D).

### Inhibition of Aurora-A reduces cellular resistance to cisplatin

**Figure 5:**
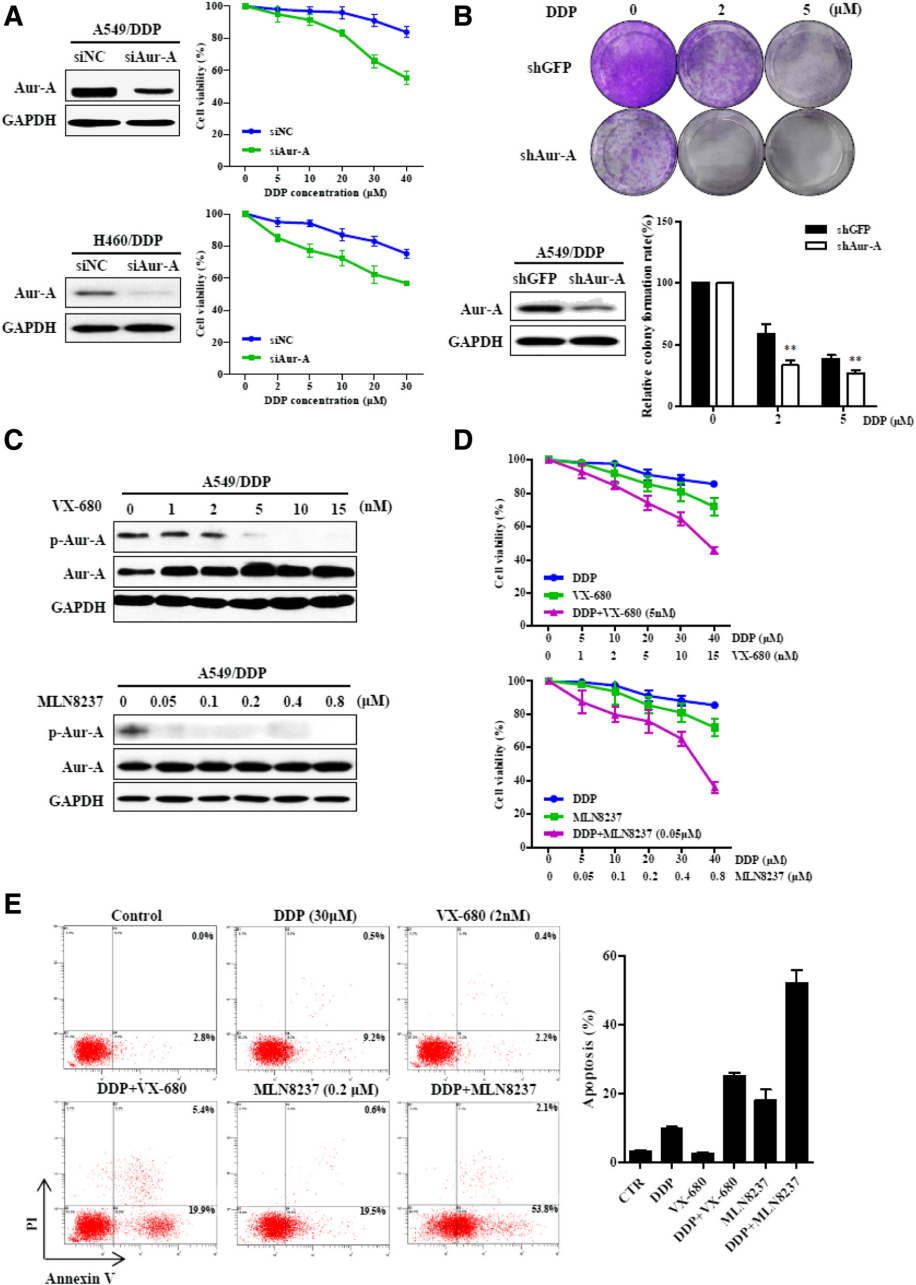
**Inhibition of Aurora-A reduces cellular resistance to cisplatin. (A)** A549/DDP and H460/DDP cells were transfected with negative control (NC) or Aurora-A siRNA for 48 h and subjected to western blotting (left panel). RNAi-treated cells were incubated with indicated concentrations of DDP for 24 h, and were harvested for MTT assays (right panel). **(B)** A549/DDP cells expressing Aurora-A shRNA or GFP shRNA (control) under Tet-inducible system were treated cells with doxycycline (Dox, 1 μg/ml) for 48 h and harvested for western blotting analysis. Cells were subjected to colony formation assay in the presence of Dox (1 μg/ml) and DDP (0, 2, 5 μM) (n = 3), **p < 0.01, two-tailed Student’s *t*-tests. **(C)** A549/DDP cells treated with increasing doses of VX-680, MLN8237or DMSO (control) for 24 h were lysed and subjected to western blotting. **(D)** A549/DDP cells were treated with DDP or VX-680, or DDP in combination with VX-680 at the indicated concentrations for 24 h, and cellular viability was assessed by MTT assay (upper panel). A549/DDP cells were treated with DDP or MLN8237, or DDP in combination with MLN8237 for 24 h, and then incubated in fresh medium for another 24 h and subjected to MTT assay (lower panel). **(E)** A549/DDP cells with indicated treatment for 24 h were subjected to annexin V-FITC and PI staining. Data were mean ± SD (n = 3).

We knocked down Aurora-A expression in A549/DDP and H460/DDP cells by siRNA (Figure 5A, left panel), and found that it resulted in a significantly enhanced sensitivity to cisplatin (Figure 5A, right panel). Colony formation assays showed that down-regulation of Aurora-A by Tet-inducible RNAi suppressed proliferation of A549/DDP cells severely in the presence of cisplatin (Figure 5B). We then used VX-680, a small-molecule inhibitor of the Aurora kinases, and Aurora-A kinase specific inhibitor MLN8237 to determine whether inhibition of Aurora-A activity would induce the similar effect. As expected, incubation of A549/DDP cells with increasing doses of VX-680 or MLN8237 led to decrease in Aurora-A phosphorylation at Thr288 (Figure 5C). Phosphorylation inhibition was also observed in histone H3 at Ser10 (Additional file 1: Figure S4). A549/DDP and H460/DDP cells were treated with single-agent DDP, VX-680, MLN8237, or DDP in combination with VX-680 or MLN8237. The results showed that the cytotoxicity effect of DDP, VX-680 or MLN8237 was not obvious, while combination of DDP and VX-680 or combination of DDP and MLN8237 produced significant higher cytotoxicity effects (Figure 5D; Additional file 1: Figure S5). We also assessed apoptotic cell death by annexin V-FITC staining. The combination treatment induced higher proportion of apoptosis, compared with single-agent treatment (Figure 5E).

### Inhibition of Aurora-A reverses migration ability of cisplatin-resistant cells

**Figure 6:**
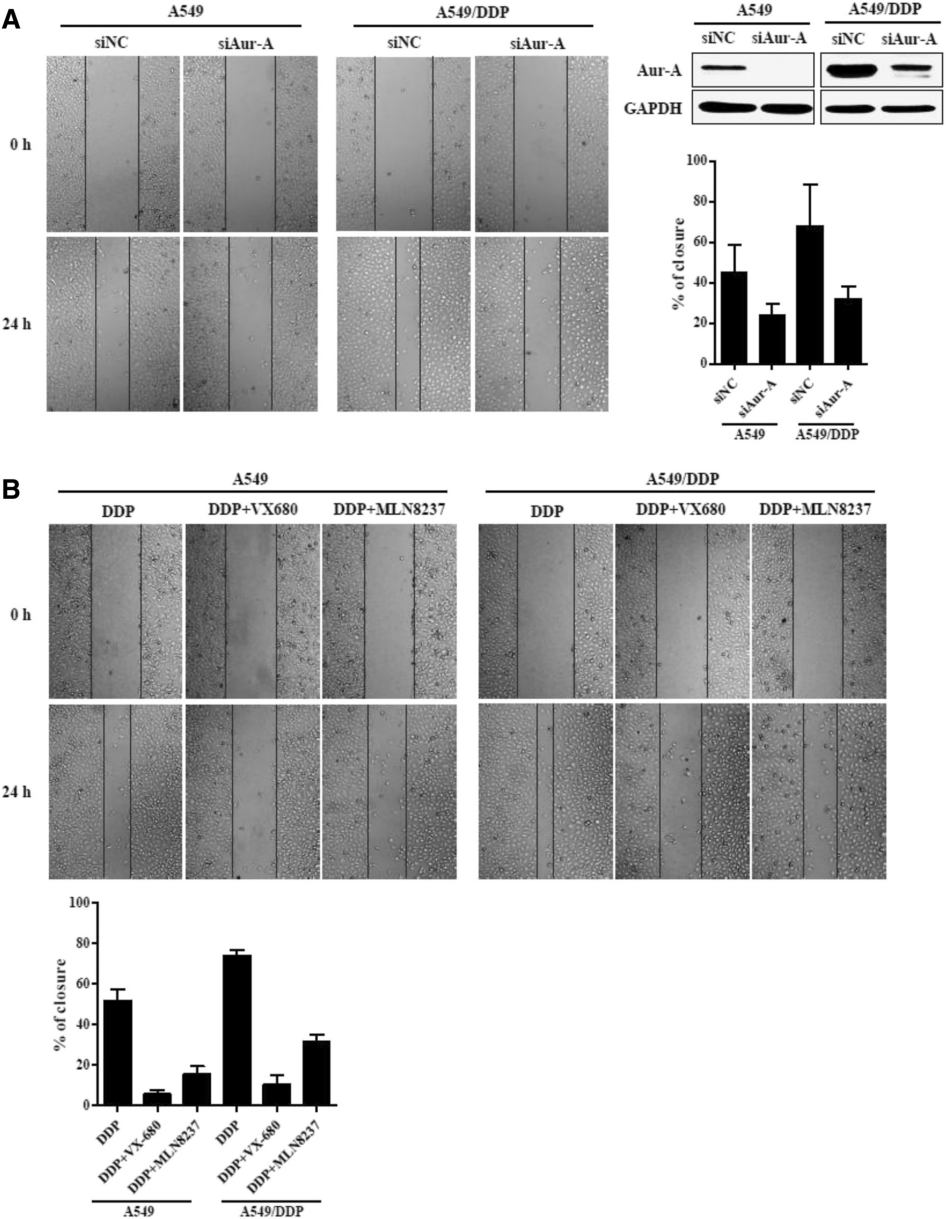
**Inhibition of Aurora-A reverses migration ability of cisplatin-resistant cells. (A)** A549 and A549/DDP cells were transfected with negative control (NC) or Aurora-A siRNA for 48 h and subjected to western blotting. Cells transfected with siRNA were treated with or without DDP (10 μM), and the cell migration ability was examined by wound healing assay. Images were captured at the time points as indicated. **(B)** A549 and A549/DDP cells were treated with DDP at 0 (control) and 10 μM, in combination with or without VX-680 (5 nM) or MLN8237 (0.2 μM). Then the cells were subjected to wound healing assay and relative wound closure quantified.

Given the association between Aurora-A expression levels and metastasis in NSCLC patients, we sought to identify whether inhibition of Aurora-A could reverse migration ability of cisplatin-resistant cells. We employed wound-healing assay to evaluate the effect of repression of Aurora-A on cell migration. As shown in Figure 6A, with DDP treatment, the migration ability of A549/DDP cells was obviously higher than A549 cells, and which was effectively inhibited by lack of Aurora-A expression. Similar results were observed by using Aurora kinase inhibitor VX-680 or MLN8237 (Figure 6B).

## Discussion

Platinum-based chemotherapy is commonly employed for the treatment of NSCLC. Cisplatin exerts its anticancer effect via the induction of mitochondrial apoptosis as a result of lethal DNA damage. Despite a consistent rate of initial responses, cisplatin treatment often results in the development of chemoresistance and numerous mechanisms contribute to cisplatin resistance. In this study, our data indicated that Aurora-A overexpression was associated with poor survival in NSCLC patients treated with cisplatin-based chemotherapy. Aurora-A overexpression decreased the sensitivity of lung cancer cells to cisplatin in vitro, and Aurora-A inhibition enhanced cisplatin-induced apoptosis. Additionally, Aurora-A promotes the migration in cisplatin-resistanct NSCLC cells, as well as the metastasis in NSCLC patients.

Aurora-A amplification and overexpression have been reported to be significantly associated with aneuploidy, high tumor grade, increased invasiveness and poor prognosis [13,29,30]. Due to dysregulation of the spindle checkpoint, Aurora-A has been implicated in conferring resistance to chemotherapy in cancer cells. As the underlying mechanism of Aurora-A involving in chemoresistance, it remains complicated and varies in various types of human cancers. Elevated Aurora-A expression confers resistance to apoptosis induced by chemotherapeutic agents [16,31,32]. Inhibition of Aurora-A in cells expressing mutant JAK2 abolishes the resistance to cisplatin [33], and enhances cisplatin-induced cell death in esophageal carcinoma cells [34,35]. It has been reported that Aurora-A induces cisplatin chemoresistance by inhibition of p53, leading to down-regulation of PTEN and activation of Akt in human ovarian cancer cells [36]. Some studies show that Aurora-A promotes chemotherapeutic drugs resistance via a NF-κB signaling pathway in p53 knockdown lung cancer cells [37].

Chemoresistance has been reported to correlate closely with metastasis in human cancer patients. Metastatic tumors are invariably more resistant to the chemotherapy compared to primary tumors as evidenced by the marked decrease of chemotherapy response rate in metastatic settings [38,39]. Moreover, recent studies have shown a link between chemotherapy resistance and the EMT phenotype [40-42]. For example, resistance to cisplatin was observed in cell lines undergoing EMT [43,44]. EMT is a process by which epithelial cells undergo transition to a mesenchymal phenotype, with increasing motility and invasive capacity. EMT increased malignant potential and reduced sensitivity to cisplatin and paclitaxel in NSCLC cells. Therapeutic combinations using EMT-signaling inhibitors may be needed to circumvent the resistance of some types of cancer to chemotherapy [45,46].

Previously, we found that Aurora-A promoted EMT and invasion in nasopharyngeal carcinoma mediated by mitogen-activated protein kinase (MAPK) phosphorylation [47], and increased laryngeal squamous cell carcinoma (LSCC) cell growth and migration mediated by activation of Akt1 [17]. More recently, we further demonstrated that Aurora-A overexpression enhanced breast cancer cell migration by activating the cofilin-F-actin pathway [18] and predicted an inferior prognosis of triple negative breast cancer (TNBC) [48]. Combined with our previous results, the presents study further support that contribution of Aurora-A overexpression to the cisplatin resistance and the poor prognosis pattern in NSCLC patients treated with cisplatin-based therapy may be ascribed to the high proliferation- and metastasis-promoting function of Aurora-A. Aurora-A may also increase the risk of metastatic recurrence against chemotherapy, although this was not proven directly in our current study due to difficulty in segregating the two functions (chemoresistance and migration). The mechanism of regulation of downstream factors by Aurora-A to clarify this link and interaction will be important matters to be addressed by future studies.

## Conclusion

In summary, our data suggest that increased expression of Aurora-A is related to cisplatin resistance and lymphatic metastasis in NSCLC and contributes to a poor prognostic phenotype. Aurora-A could be a potential cisplatin-resistance target in NSCLC, and inhibition of Aurora-A kinase is a promising regimen to overcome cisplatin resistance in NSCLC patients.

## Abbreviations

NSCLC: Non-small cell lung cancer; DDP: Cisplatin; ROC: Receiver operating characteristic; OS: Overall survival; PFS: Progression-free survival; EMT: Epithelial-mesenchymal transition.

## Competing interests

The authors declare that they have no conflict of interest.

## Authors’ contributions

QL and AWL carried out and coordinated the study. JX, CFY and WHZ performed the experiments and analyzed the data. CFY and WHZ wrote the paper. YMQ, YZ and SWW participated in manuscript revising and editing. All authors read and approved the final manuscript.

## Supplementary Material

Additional file 1: Figure S1. Detection of the specificity for antibody against Aurora-A. A549/DDP cells were lysed and subjected to western blotting. **Figure S2.** Receiver operating characteristic (ROC) curves analysis of Aurora-A cutoff score in the training set. (A) Aurora-A cutoff point for overall survival in the training set. (B) Aurora-A cutoff point for progression-free survival in the training set. At each immunohistochemical score, the sensitivity and specificity for the outcome being studied was plotted, thus generating a ROC curve. Aurora-A cutoff score for overall survival, progression-free survival was 3.4 and 3.2 respectively. **Figure S3.** Growth curve of A549 and A549/DDP cells. The growth curve of A549 and A549/DDP cells was analyzed using cell number. Data were mean ± SD of 3 independent experiments; ***p < 0.001, two-way ANOVA analysis. **Figure S4.** Analysis of histone H3 phosphorylation (Ser 10) level. A549/DDP cells treated with increasing doses of VX-680 for 24 h were lysed and subjected to western blotting. **Figure S5.** Inhibition of Aurora-A reduces H460/DDP cells resistance to cisplatin. (A) H460/DDP cells were treated with DDP or VX-680, or DDP in combination with VX-680 at the indicated concentrations for 24 h, and cellular viability was assessed by MTT assay (left panel). (B) H460/DDP cells were treated with DDP or MLN8237, or DDP in combination with MLN8237 for 24 h, and then incubated in fresh medium for another 24 h and subjected to MTT assay (right panel). Click here for file

Additional file 2: Table S1. Results of univariate and multivariate Cox proportional-hazards analysis in the overall patient for progression-free survival. Click here for file

## References

[B1] Jemal A Bray F Center MM Ferlay J Ward E Forman D Global cancer statistics CA Cancer J Clin 2011 61 69 90 21296855 10.3322/caac.20107

[B2] Ettinger DS Bepler G Bueno R Chang A Chang JY Chirieac LR D’Amico TA Demmy TL Feigenberg SJ Grannis FW Jr Jahan T Jahanzeb M Kessinger A Komaki R Kris MG Langer CJ Le QT Martins R Otterson GA Robert F Sugarbaker DJ Wood DE National Comprehensive Cancer Network (NCCN) Non-small cell lung cancer clinical practice guidelines in oncology J Natl Compr Canc Netw 2006 4 548 582 16813724 10.6004/jnccn.2006.0046

[B3] Spira A Ettinger DS Multidisciplinary management of lung cancer N Engl J Med 2004 350 379 392 14736930 10.1056/NEJMra035536

[B4] Mok TSK Ramalingam SS Maintenance therapy in nonsmall-cell lung cancer Cancer 2009 115 5143 5154 19658185 10.1002/cncr.24563

[B5] D’Amato TA Landreneau RJ McKenna RJ Santos RS Parker RJ Prevalence of in vitro extreme chemotherapy resistance in resected nonsmall-cell lung cancer Ann Thorac Surg 2006 81 440 446 discussion 446–447 16427828 10.1016/j.athoracsur.2005.08.037

[B6] Wang D Lippard SJ Cellular processing of platinum anticancer drugs Nat Rev Drug Discov 2005 4 307 320 15789122 10.1038/nrd1691

[B7] Friboulet L Olaussen KA Pignon JP Shepherd FA Tsao MS Graziano S Kratzke R Douillard JY Seymour L Pirker R Filipits M André F Solary E Ponsonnailles F Robin A Stoclin A Dorvault N Commo F Adam J Vanhecke E Saulnier P Thomale J Le Chevalier T Dunant A Rousseau V Le Teuff G Brambilla E Soria JC ERCC1 isoform expression and DNA repair in non-small-cell lung cancer N Engl J Med 2013 368 1101 1110 23514287 10.1056/NEJMoa1214271 PMC4054818

[B8] Kang CH Jang BG Kim DW Chung DH Kim YT Jheon S Sung SW Kim JH The prognostic significance of ERCC1, BRCA1, XRCC1, and betaIII-tubulin expression in patients with non-small cell lung cancer treated by platinum- and taxane-based neoadjuvant chemotherapy and surgical resection Lung Cancer 2010 68 478 483 19683826 10.1016/j.lungcan.2009.07.004

[B9] Castro MA Dal-Pizzol F Zdanov S Soares M Muller CB Lopes FM Zanotto-Filho A da Cruz Fernandes M Moreira JC Shacter E Klamt F CFL1 expression levels as a prognostic and drug resistance marker in nonsmall cell lung cancer Cancer 2010 116 3645 3655 20564088 10.1002/cncr.25125 PMC2910822

[B10] Marumoto T Zhang D Saya H Aurora-A - a guardian of poles Nat Rev Cancer 2005 5 42 50 15630414 10.1038/nrc1526

[B11] Bischoff JR Anderson L Zhu Y Mossie K Ng L Souza B Schryver B Flanagan P Clairvoyant F Ginther C Chan CS Novotny M Slamon DJ Plowman GD A homologue of Drosophila aurora kinase is oncogenic and amplified in human colorectal cancers EMBO J 1998 17 3052 3065 9606188 10.1093/emboj/17.11.3052 PMC1170645

[B12] Tanaka T Kimura M Matsunaga K Fukada D Mori H Okano Y Centrosomal kinase AIK1 is overexpressed in invasive ductal carcinoma of the breast Cancer Res 1999 59 2041 2044 10232583

[B13] Gritsko TM Coppola D Paciga JE Yang L Sun M Shelley SA Fiorica JV Nicosia SV Cheng JQ Activation and overexpression of centrosome kinase BTAK/Aurora-A in human ovarian cancer Clin Cancer Res 2003 9 1420 1426 12684414

[B14] Li D Zhu J Firozi PF Abbruzzese JL Evans DB Cleary K Friess H Sen S Overexpression of oncogenic STK15/BTAK/Aurora A kinase in human pancreatic cancer Clin Cancer Res 2003 9 991 997 12631597

[B15] Tanaka E Hashimoto Y Ito T Okumura T Kan T Watanabe G Imamura M Inazawa J Shimada Y The clinical significance of Aurora-A/STK15/BTAK expression in human esophageal squamous cell carcinoma Clin Cancer Res 2005 11 1827 1834 15756006 10.1158/1078-0432.CCR-04-1627

[B16] Anand S Penrhyn-Lowe S Venkitaraman AR AURORA-A amplification overrides the mitotic spindle assembly checkpoint, inducing resistance to Taxol Cancer Cell 2003 3 51 62 12559175 10.1016/s1535-6108(02)00235-0

[B17] Guan Z Wang XR Zhu XF Huang XF Xu J Wang LH Wan XB Long ZJ Liu JN Feng GK Huang W Zeng YX Chen FJ Liu Q Aurora-A, a negative prognostic marker, increases migration and decreases radiosensitivity in cancer cells Cancer Res 2007 67 10436 10444 17974987 10.1158/0008-5472.CAN-07-1379

[B18] Wang LH Xiang J Yan M Zhang Y Zhao Y Yue CF Xu J Zheng FM Chen JN Kang Z Chen TS Xing D Liu Q The mitotic kinase Aurora-A induces mammary cell migration and breast cancer metastasis by activating the Cofilin-F-actin pathway Cancer Res 2010 70 9118 9128 21045147 10.1158/0008-5472.CAN-10-1246

[B19] Katayama H Sen S Aurora kinase inhibitors as anticancer molecules Biochim Biophys Acta 2010 1799 829 839 20863917 10.1016/j.bbagrm.2010.09.004 PMC4501772

[B20] Matulonis UA Sharma S Ghamande S Gordon MS Del Prete SA Ray-Coquard I Kutarska E Liu H Fingert H Zhou X Danaee H Schilder RJ Phase II study of MLN8237 (alisertib), an investigational Aurora A kinase inhibitor, in patients with platinum-resistant or -refractory epithelial ovarian, fallopian tube, or primary peritoneal carcinoma Gynecol Oncol 2012 127 63 69 22772063 10.1016/j.ygyno.2012.06.040

[B21] Friedberg JW Mahadevan D Cebula E Persky D Lossos I Agarwal AB Jung J Burack R Zhou X Leonard EJ Fingert H Danaee H Bernstein SH Phase II study of alisertib, a selective Aurora A kinase inhibitor, in relapsed and refractory aggressive B- and T-cell non-Hodgkin lymphomas J Clin Oncol 2014 32 44 50 24043741 10.1200/JCO.2012.46.8793 PMC3867644

[B22] Edge SB Compton CC The American Joint Committee on Cancer: the 7th edition of the AJCC cancer staging manual and the future of TNM Ann Surg Oncol 2010 17 1471 1474 20180029 10.1245/s10434-010-0985-4

[B23] Li JJ Ding Y Li DD Peng RQ Feng GK Zeng YX Zhu XF Zhang XS The overexpression of ERCC-1 is involved in the resistance of lung cancer cells to cetuximab combined with DDP Cancer Biol Ther 2009 8 1914 1921 20009541 10.4161/cbt.8.20.9439

[B24] Yang H Fu JH Hu Y Huang WZ Zheng B Wang G Zhang X Wen J Influence of SiRNA targeting survivin on chemosensitivity of H460/cDDP lung cancer cells J Int Med Res 2008 36 734 747 18652770 10.1177/147323000803600416

[B25] Yan M Zhang Y He B Xiang J Wang ZF Zheng FM Xu J Chen MY Zhu YL Wen HJ Wan XB Yue CF Yang N Zhang W Zhang JL Wang J Wang Y Li LH Zeng YX Lam EW Hung MC Liu Q IKKalpha restoration via EZH2 suppression induces nasopharyngeal carcinoma differentiation Nat Commun 2014 5 3661 24739462 10.1038/ncomms4661

[B26] Cheng AL Huang WG Chen ZC Peng F Zhang PF Li MY Li F Li JL Li C Yi H Yi B Xiao ZQ Identification of novel nasopharyngeal carcinoma biomarkers by laser capture microdissection and proteomic analysis Clin Cancer Res 2008 14 435 445 18223218 10.1158/1078-0432.CCR-07-1215

[B27] Zhou WH Tang F Xu J Wu X Yang SB Feng ZY Ding YG Wan XB Guan Z Li HG Lin DJ Shao CK Liu Q Low expression of Beclin 1, associated with high Bcl-xL, predicts a malignant phenotype and poor prognosis of gastric cancer Autophagy 2012 8 389 400 22240664 10.4161/auto.18641

[B28] Zlobec I Steele R Terracciano L Jass JR Lugli A Selecting immunohistochemical cut-off scores for novel biomarkers of progression and survival in colorectal cancer J Clin Pathol 2007 60 1112 1116 17182662 10.1136/jcp.2006.044537 PMC2014838

[B29] Zhou H Kuang J Zhong L Kuo WL Gray JW Sahin A Brinkley BR Sen S Tumour amplified kinase STK15/BTAK induces centrosome amplification, aneuploidy and transformation Nat Genet 1998 20 189 193 9771714 10.1038/2496

[B30] Dar AA Goff LW Majid S Berlin J El-Rifai W Aurora kinase inhibitors–rising stars in cancer therapeutics? Mol Cancer Ther 2010 9 268 278 20124450 10.1158/1535-7163.MCT-09-0765 PMC2820587

[B31] Cammareri P Scopelliti A Todaro M Eterno V Francescangeli F Moyer MP Agrusa A Dieli F Zeuner A Stassi G Aurora-a is essential for the tumorigenic capacity and chemoresistance of colorectal cancer stem cells Cancer Res 2010 70 4655 4665 20460511 10.1158/0008-5472.CAN-09-3953

[B32] Scharer CD Laycock N Osunkoya AO Logani S McDonald JF Benigno BB Moreno CS Aurora kinase inhibitors synergize with paclitaxel to induce apoptosis in ovarian cancer cells J Transl Med 2008 6 79 19077237 10.1186/1479-5876-6-79 PMC2614415

[B33] Sumi K Tago K Kasahara T Funakoshi-Tago M Aurora kinase A critically contributes to the resistance to anti-cancer drug cisplatin in JAK2 V617F mutant-induced transformed cells FEBS Lett 2011 585 1884 1890 21557940 10.1016/j.febslet.2011.04.068

[B34] Sehdev V Peng D Soutto M Washington MK Revetta F Ecsedy J Zaika A Rau TT Schneider-Stock R Belkhiri A El-Rifai W The aurora kinase A inhibitor MLN8237 enhances cisplatin-induced cell death in esophageal adenocarcinoma cells Mol Cancer Ther 2012 11 763 774 22302096 10.1158/1535-7163.MCT-11-0623 PMC3297687

[B35] Wang XX Liu R Jin SQ Fan FY Zhan QM Overexpression of Aurora-A kinase promotes tumor cell proliferation and inhibits apoptosis in esophageal squamous cell carcinoma cell line Cell Res 2006 16 356 366 16617331 10.1038/sj.cr.7310046

[B36] Yang H He L Kruk P Nicosia SV Cheng JQ Aurora-A induces cell survival and chemoresistance by activation of Akt through a p53-dependent manner in ovarian cancer cells Int J Cancer 2006 119 2304 2312 16894566 10.1002/ijc.22154

[B37] Sun C Chan F Briassouli P Linardopoulos S Aurora kinase inhibition downregulates NF-kappaB and sensitises tumour cells to chemotherapeutic agents Biochem Biophys Res Commun 2007 352 220 225 17113039 10.1016/j.bbrc.2006.11.004

[B38] Acharyya S Oskarsson T Vanharanta S Malladi S Kim J Morris Patrick G Manova-Todorova K Leversha M Hogg N Seshan Venkatraman E Norton L Brogi E Massague J A CXCL1 paracrine network links cancer chemoresistance and metastasis Cell 2012 150 165 178 22770218 10.1016/j.cell.2012.04.042 PMC3528019

[B39] Wei Y Hu G Kang Y Metadherin as a link between metastasis and chemoresistance Cell Cycle 2009 8 2132 2133 19556872

[B40] Holohan C Van Schaeybroeck S Longley DB Johnston PG Cancer drug resistance: an evolving paradigm Nat Rev Cancer 2013 13 714 726 24060863 10.1038/nrc3599

[B41] Arumugam T Ramachandran V Fournier KF Wang H Marquis L Abbruzzese JL Gallick GE Logsdon CD McConkey DJ Choi W Epithelial to mesenchymal transition contributes to drug resistance in pancreatic cancer Cancer Res 2009 69 5820 5828 19584296 10.1158/0008-5472.CAN-08-2819 PMC4378690

[B42] Nurwidya F Takahashi F Murakami A Takahashi K Epithelial mesenchymal transition in drug resistance and metastasis of lung cancer Cancer Res Treat 2012 44 151 156 23091440 10.4143/crt.2012.44.3.151 PMC3467417

[B43] Wang Z Li Y Kong D Banerjee S Ahmad A Azmi AS Ali S Abbruzzese JL Gallick GE Sarkar FH Acquisition of epithelial-mesenchymal transition phenotype of gemcitabine-resistant pancreatic cancer cells is linked with activation of the notch signaling pathway Cancer Res 2009 69 2400 2407 19276344 10.1158/0008-5472.CAN-08-4312 PMC2657919

[B44] Kajiyama H Shibata K Terauchi M Yamashita M Ino K Nawa A Kikkawa F Chemoresistance to paclitaxel induces epithelial-mesenchymal transition and enhances metastatic potential for epithelial ovarian carcinoma cells Int J Oncol 2007 31 277 283 17611683

[B45] Shintani Y Okimura A Sato K Nakagiri T Kadota Y Inoue M Sawabata N Minami M Ikeda N Kawahara K Matsumoto T Matsuura N Ohta M Okumura M Epithelial to mesenchymal transition is a determinant of sensitivity to chemoradiotherapy in non-small cell lung cancer Ann Thorac Surg 2011 92 1794 1804 discussion 1804 22051275 10.1016/j.athoracsur.2011.07.032

[B46] Voulgari A Pintzas A Epithelial-mesenchymal transition in cancer metastasis: mechanisms, markers and strategies to overcome drug resistance in the clinic Biochim Biophys Acta 2009 1796 75 90 19306912 10.1016/j.bbcan.2009.03.002

[B47] Wan XB Long ZJ Yan M Xu J Xia LP Liu L Zhao Y Huang XF Wang XR Zhu XF Hong MH Liu Q Inhibition of Aurora-A suppresses epithelial-mesenchymal transition and invasion by downregulating MAPK in nasopharyngeal carcinoma cells Carcinogenesis 2008 29 1930 1937 18667445 10.1093/carcin/bgn176

[B48] Xu J Wu X Zhou WH Liu AW Wu JB Deng JY Yue CF Yang SB Wang J Yuan ZY Liu Q Aurora-A identifies early recurrence and poor prognosis and promises a potential therapeutic target in triple negative breast cancer PLoS One 2013 8 e56919 23437271 10.1371/journal.pone.0056919 PMC3577665

