# Supplementary figures and images for: Aurora-A contributes to cisplatin resistance and lymphatic metastasis in non-small cell lung cancer and predicts poor prognosis
Source: J Transl Med. 2014 Jul 31;12:200. doi: 10.1186/1479-5876-12-200 (PMC4237886; doi:10.1186/1479-5876-12-200)

**Figure S1**

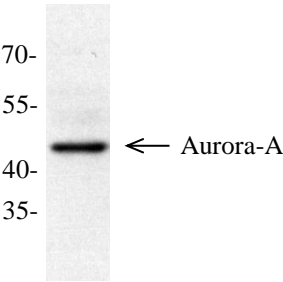

Figure S2

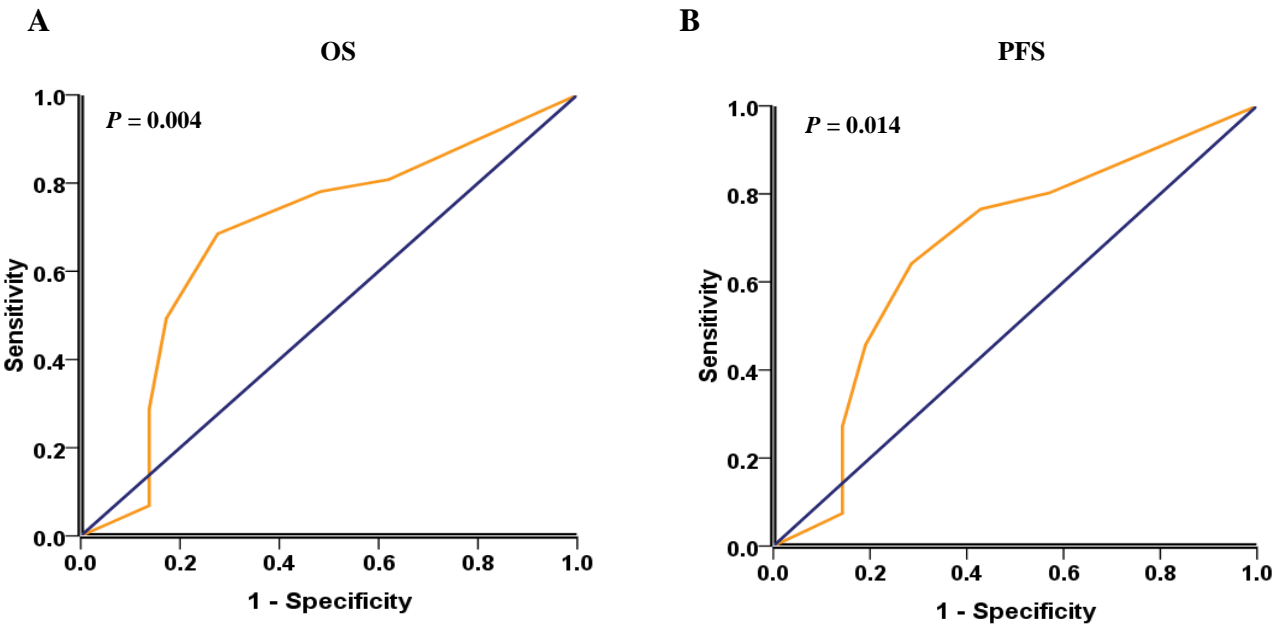

Figure S3

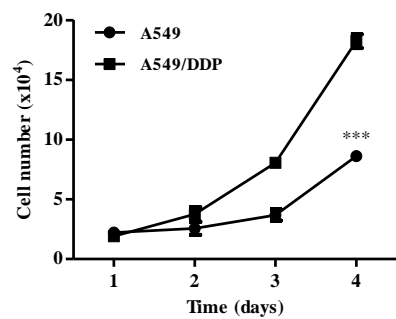

Figure S4

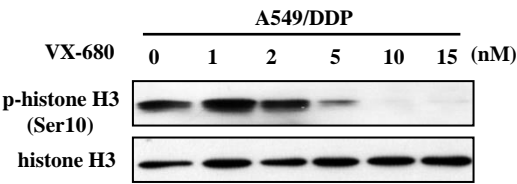

Figure S5

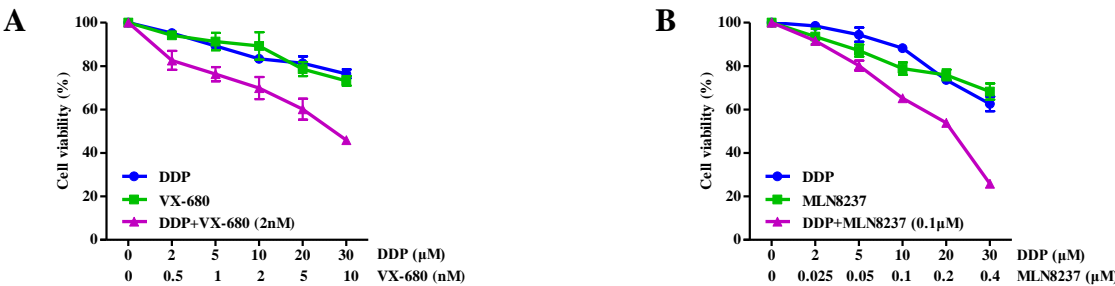

Supplement: Additional file 1: Figure S1. — Detection of the specificity for antibody against Aurora-A. A549/DDP cells were lysed and subjected to western blotting. Figure S2. Receiver operating characteristic (ROC) curves analysis of Aurora-A cutoff score in the training set. (A) Aurora-A cutoff point for overall survival in the training set. (B) Aurora-A cutoff point for progression-free survival in the training set. At each immunohistochemical score, the sensitivity and specificity for the outcome being studied was plotted, thus generating a ROC curve. Aurora-A cutoff score for overall survival, progression-free survival was 3.4 and 3.2 respectively. Figure S3. Growth curve of A549 and A549/DDP cells. The growth curve of A549 and A549/DDP cells was analyzed using cell number. Data were mean ± SD of 3 independent experiments; ***p < 0.001, two-way ANOVA analysis. Figure S4. Analysis of histone H3 phosphorylation (Ser 10) level. A549/DDP cells treated with increasing doses of VX-680 for 24 h were lysed and subjected to western blotting. Figure S5. Inhibition of Aurora-A reduces H460/DDP cells resistance to cisplatin. (A) H460/DDP cells were treated with DDP or VX-680, or DDP in combination with VX-680 at the indicated concentrations for 24 h, and cellular viability was assessed by MTT assay (left panel). (B) H460/DDP cells were treated with DDP or MLN8237, or DDP in combination with MLN8237 for 24 h, and then incubated in fresh medium for another 24 h and subjected to MTT assay (right panel). [file 1479-5876-12-200-S1.pdf]
