# Supplementary material for: Aurora-A contributes to cisplatin resistance and lymphatic metastasis in non-small cell lung cancer and predicts poor prognosis
Source: J Transl Med. 2014 Jul 31;12:200. doi: 10.1186/1479-5876-12-200 (PMC4237886; doi:10.1186/1479-5876-12-200)
Supplement: Additional file 2: Table S1. — Results of univariate and multivariate Cox proportional-hazards analysis in the overall patient for progression-free survival. [file 1479-5876-12-200-S2.docx]

**Table S1.** Results of univariate and multivariate Cox proportional-hazards analysis in the overall patient for progression-free survival.

|  |  | **For progression-free survival** | | | |  |
| --- | --- | --- | --- | --- | --- | --- |
| **Variable** |  | **Univariate analysis** |  |  | **Multivariate analysis** |  |
|  | **Hazard Ratio** | **95%**  **confidence interval** | ***P*** | **Hazard Ratio** | **95%**  **confidence interval** | ***P*** |
| **Age (years)**  **≥ 60.00 (VS. < 60.0)** | 1.309 | (0.656 to 1.646) | 0.871 | 1.241 | (0.746 to 2.062) | 0.405 |
| **Gender**  **Male (VS. Female)** | 1.264 | (0.778 to 2.055) | 0.344 | 1.536 | (0.691 to 3.414) | 0.899 |
| **Smoking history**  **Yes (VS. No )** | 1.414 | (0.905 to 2.209) | 0.128 | 1.216 | (0.580 to 2.551) | 0.604 |
| **CEA (ng/ml)**  **> 5 ( VS. ≤ 5)** | 1.996 | (1.275 to 3.126) | 0.003 | 2.169 | (1.371 to 3.432) | 0.001 |
| **Initial clinical stage** |  |  |  |  |  |  |
| I | 0.367 | (0.157 to 0.857) | 0.021 | 0.592 | (0.152 to 2.311) | 0.451 |
| II | 0.480 | (0.270 to 0.852) | 0.012 | 0.827 | (0.361 to 1.896) | 0.654 |
| III | 1 | 1 |  | 1 | 1 |  |
| **Histology** |  |  |  |  |  |  |
| Squamous cell carcinoma | 0.758 | (0.185 to 3.101) | 0.700 | 2.126 | (0.466 to 9.706) | 0.330 |
| Adenocarcinoma | 0.776 | (0.167 to 3.606) | 0.746 | 1.959 | (0.365 to 10.507) | 0.432 |
| Adenosquamous cell carcinomas | 1 | 1 |  | 1 | 1 |  |
| **Differentiatiation** |  |  |  |  |  |  |
| Highly | 0.573 | (0.287 to 1.145) | 0.115 | 0.959 | (0.447 to 2.060) | 0.915 |
| Moderately | 0.848 | (0.526 to 1.367) | 0.498 | 1.017 | (0.605 to 1.710) | 0.948 |
| Poorly | 1 | 1 |  | 1 | 1 |  |
| **Tumor stage T4 + T3 (VS. T2 + T1)** | 1.591 | (1.017 to 2.490) | 0.042 | 1.937 | (1.224 to 3.067) | 0.005 |
| **Lymph node metastasis Positive (VS. Negative)** | 1.758 | (1.025 to 3.015) | 0.040 | 1.170 | (0.444 to 3.086) | 0.751 |
| **Aurora-A**  **High (VS. Low)** | 3.046 | (1.897 to 4.889) | 0.000 | 3.360 | (2.066 to 5.466) | 0.000 |
